# Integrating Transcriptomics with Metabolic Modeling Predicts Biomarkers and Drug Targets for Alzheimer's Disease

**DOI:** 10.1371/journal.pone.0105383

**Published:** 2014-08-15

**Authors:** Shiri Stempler, Keren Yizhak, Eytan Ruppin

**Affiliations:** 1 The Sackler School of Medicine – Tel Aviv University, Tel Aviv, Israel; 2 The Blavatnik School of Computer Science – Tel Aviv University, Tel Aviv, Israel; Virginia Commonwealth University, United States of America

## Abstract

Accumulating evidence links numerous abnormalities in cerebral metabolism with the progression of Alzheimer's disease (AD), beginning in its early stages. Here, we integrate transcriptomic data from AD patients with a genome-scale computational human metabolic model to characterize the altered metabolism in AD, and employ state-of-the-art metabolic modelling methods to predict metabolic biomarkers and drug targets in AD. The metabolic descriptions derived are first tested and validated on a large scale versus existing AD proteomics and metabolomics data. Our analysis shows a significant decrease in the activity of several key metabolic pathways, including the carnitine shuttle, folate metabolism and mitochondrial transport. We predict several metabolic biomarkers of AD progression in the blood and the CSF, including succinate and prostaglandin D2. Vitamin D and steroid metabolism pathways are enriched with predicted drug targets that could mitigate the metabolic alterations observed. Taken together, this study provides the first network wide view of the metabolic alterations associated with AD progression. Most importantly, it offers a cohort of new metabolic leads for the diagnosis of AD and its treatment.

## Introduction

Alzheimer's disease (AD) is the most common form of dementia. It is estimated that AD affects more than 35 million patients worldwide and its incidence is expected to increase with the aging of the population. Although extensive investigations of AD have taken place over the past few decades, its pathogenesis has yet to be elucidated. Currently no treatment is available to prevent or halt the progression of AD. Moreover, the clinical diagnosis of AD is not possible until a patient reaches the dementia phase of the disease [1]. A more accurate and earlier diagnosis of AD could enable the use of potential disease-modifying drugs and thus, there is a need for biological markers for the early stages of AD [2].

Metabolic alterations have been proposed to be involved in AD from the early stages of the disease [3]. Increasing evidence indicates an antecedent and potentially causal role of brain hypometabolism in AD pathogenesis [4]. Perturbations in mitochondrial function have long been observed in AD patients, including decreased activity of key mitochondrial enzymes [4], [5]. Consequently, ATP production and oxygen consumption become impaired [6]. Impaired glucose transport has also been reported in AD brains. Moreover, there is a link between cholesterol turnover and neurodegenerative diseases and hypercholesterolemia has been proposed as a risk factor for AD [7]. However, the relationship between cholesterol levels and the clinical manifestation of dementia remains unclear [8]. There is also a debate regarding the role of certain vitamins such as vitamin D and folic acid in the pathogenesis of AD [9], [10]_ENREF_14. Clearly from all of this mounting evidence, multiple metabolic pathways may play a key role in AD's progression.

Recent studies of gene expression from brains of AD patients further point to the strong association between metabolic alterations and AD, already from the early stages of the disease [11], [12]. However, such gene expression analyses have been limited to transcriptional alterations and therefore cannot encompass the effects of putative post-transcriptional modifications that are known to play an important role in metabolism [13]. Furthermore, they do not allow the identification of biomarkers and drug targets in any direct manner. Our aim here is to go beyond these gene expression results and to elucidate the metabolic changes in AD by employing the increasingly prevalent toolkit of analysis methods provided by the emerging field of Genome-Scale Metabolic Modeling (GSMM).

GSMMs have become trusted tools in the study of metabolic networks [14], and provide a platform for interpreting omics data in a biochemically meaningful manner [15]. GSMM analysis mostly relies on constraint-based modeling (CBM), in which constraints are systematically imposed on the GSMM solution space, and the outcomes of the model are limited to physically realizable phenotypes. GSMMs have been extensively used for the study of metabolism in microorganisms and in humans both in health and disease, enabling the prediction of various metabolic phenotypes such as enzyme activities and metabolite uptake and secretion fluxes, as well as interpretation of various types of high throughput data, often yielding clinically relevant results [16]–[21]. In a recent GSMM paper studying brain metabolism, three different neuronal sub-types were reconstructed in a GSMM of brain energy metabolism [22]. Focused on the core of cerebral energy metabolism, this reconstruction has suggested that glutamate decarboxylase provides a neuroprotective effect which is correlated with the brain regional specificity of AD [22].

**Figure 1:**
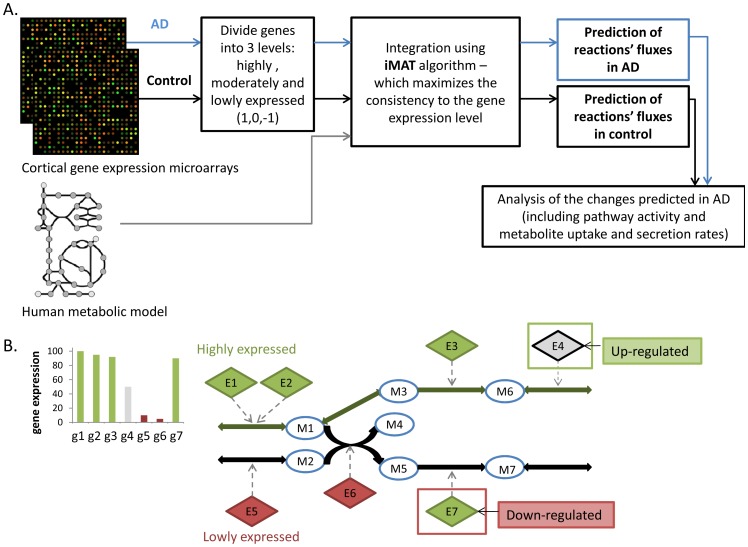
The workflow of iMAT analysis. A. First, we discretize the expression of each metabolic gene measured into 3 levels: high, moderate and low. Next, iMAT integrates these expression levels into the human metabolic model by maximizing the number of enzymes whose predicted flux activity is consistent with their expression level, yielding a prediction of the overall network flux distribution that is most consistent with the model's constraints under steady state. This analysis is done separately for the control and the AD states. B. A Toy example of the integration of the metabolic network and gene-expression by iMAT and the prediction of enzyme flux-activities (taken and modified from [18]). Circular nodes represent metabolites, solid edges represent reactions, and diamond nodes represent enzymes associated by arrows to the reactions they catalyze. Grey, red and green represent moderate, significantly low and significantly high expression of the enzyme-encoding genes, respectively. The predicted flux involving the activation of reactions is shown as green edges. Enzymes E4 and E7 are predicted to be post-transcriptionally up-regulated and down-regulated respectively.

Our investigation begins with an effort to harness GSMM to systematically describe the metabolic state in AD on a global, network level. We do this by employing a method termed integrative Metabolic Analysis Tool (iMAT), which incorporates gene expression into a GSMM to predict metabolic flux activity [18]. This method has already been shown to successfully predict tissue specific metabolic activity in several healthy human tissues, including the brain [18]. iMAT incorporates gene expression to predict global metabolic flux activity that is the most consistent with known constraints across the entire metabolic network, and reflects post transcriptional modifications that are not evident in the raw expression data (Figure 1). We utilized a relatively large dataset of gene expression microarrays from the cortex of AD patients and elderly controls [23] (including 363 samples), which we integrated with the human metabolic model to study the metabolic changes in AD. This model-based genome-scale view of AD metabolism leads to the identification of various pathways whose activities are altered significantly in AD, and importantly, are not revealed by standard pathway enrichment analysis of the raw gene expression solely, in a model-free manner. We next predict novel biomarkers for AD by comparing predicted uptake and secretion fluxes of various metabolites as the disease progresses. Finally, we predict perturbations in the metabolic network that can transform the metabolic state of AD back closer to a healthy state, highlighting new potential metabolic drug targets for AD that may work on a global, network level.

## Methods

### Datasets

The microarrays data used in this study were obtained from the Gene Expression Omnibus (GEO) site (www.ncbi.nlm.nih.gov). The first dataset contains expression data from 363 cortical samples of controls and AD patients' post-mortem brains (GSE15222) [23]. We additionally analyzed blood leukocytes gene expression that includes 3 controls, 3 AD and 3 MCI samples (GSE18309) [24]. All datasets were filtered for metabolic genes included in the human metabolic model [16].

### iMAT analysis

We first employed a discrete representation of significantly high or low enzyme-expression levels across tissues. Gene expression levels from the microarray analysis were discretized to highly (1), lowly (−1), or moderately (0) expressed, for each sample. This discretization was based on a threshold of the mean expression +0.3 SD for highly expressed genes, the mean −0.3 SD for lowly expressed genes. Genes between these thresholds were defined as 0, and the entire process is applied for each sample separately. As iMAT requires only a single such discrete representation, the final input includes only those reactions that were classified as highly/lowly expressed in at least 2/3 of the samples. The list of genes that were defined as highly and lowly expressed as input for iMAT is detailed in Table S1. In the iMAT [18] analysis, the discretized gene expression levels were incorporated into the metabolic model to predict a set of high and low activity reactions. Network integration is done by mapping the genes to the reactions according to the metabolic model, and by solving a constraint-based modeling optimization problem to find a steady-state metabolic flux distribution, following [18]. By using this CBM approach we assign permissible flux ranges to all the reactions in the network, in a way that satisfies the stoichiometric and thermodynamic constraints embedded in the model and maximizes the number of reactions whose activity is consistent with their expression state. The simulation conditions that were used were the default ones, i.e. the boundaries of the model reaction fluxes are between −1000 to 1000.

### Enrichment of metabolic pathways (gene expression and iMAT)

Based on iMAT results, which predict the activity of the reactions in the metabolic model, a hypergeometric p-value was computed for each pathway in the model for being enriched with active or inactive reactions in AD. Subsequently, for comparison of the iMAT results to the gene expression, gene expression measurements were forst translated to the reaction level using the model's gene-protein-reactions mapping, and subsequently the list of altered reactions was again analyzed for pathway enrichment in a standard manner as above. In both analyses, a correction for multiple hypotheses was done using false discovery rate (FDR) method of 0.05.

### Flux Variability Analysis (FVA)_ENREF_26 [25]

Metabolic biomarkers are predicted based on a comparison of exchange reaction intervals between the healthy case and each of the disease states. For exchange intervals A = [minA, maxA] and B = [minB, maxB] (where A and B represent the flux intervals in the control and AD stages), we define: A<B if ((minA < minB) & (maxA ≤ maxB)) | ((minA ≤ minB) & (maxA < maxB)). To consider only significant changes between exchange intervals, a difference in flux, denoted A<B, is considered only when A is at least 90% lower than B.

### Metabolic Transformation Algorithm (MTA)

The MTA algorithm gets as input gene expression levels of two metabolic states, termed source and targets states. Next, the MTA approach works to: (1) infer the most likely distribution of fluxes in the source state using iMAT; (2) identify the set of genes that their expression have significantly changed between the source and targets states, and the set of genes that their expression remain constant. Following, the algorithm searches for perturbations that can globally shift all the fluxes of the changed reactions in the right direction, while keeping the fluxes of the unchanged reaction as close as possible to their predicted source state. Finally, MTA outputs a ranked list of candidate perturbations according to their ability to result with a successful transformation, from the source to the target metabolic state^44^. The top 10% of the highest scoring reactions were used for calculation of the pathways that are enriched with predicted drug targets, as in [26].

## Results

### Network-based description of metabolic alterations in AD and their large-scale validation

Our first goal in this study was to uncover the major metabolic alterations that differentiate AD-afflicted brains from healthy ones. As transcriptional regulation plays a major role in controlling metabolic functions [13], and there is a large body of transcriptome data available for study, we approached this problem using iMAT, a computational method to systematically predict metabolic behavior by incorporating gene expression data into a GSMM [18]. We started by integrating an expression dataset of metabolic genes from the cortex of both healthy and AD elderly subjects [23] into the human metabolic model (see Methods, Figure 1). To account for metabolic flux activity that is not reflected in the mRNA expression data, iMAT considers the mRNA levels as cues for the likelihood that the enzyme in question carries a metabolic flux in its associated reaction(s), and then leverages the GSMM to accumulate these cues into a global flux behavior that is stochiometrically consistent and maintains mass balance across the entire network [27]. Hence, iMAT predicts a feasible flux distribution that best agrees with the gene expression data.

**Figure 2:**
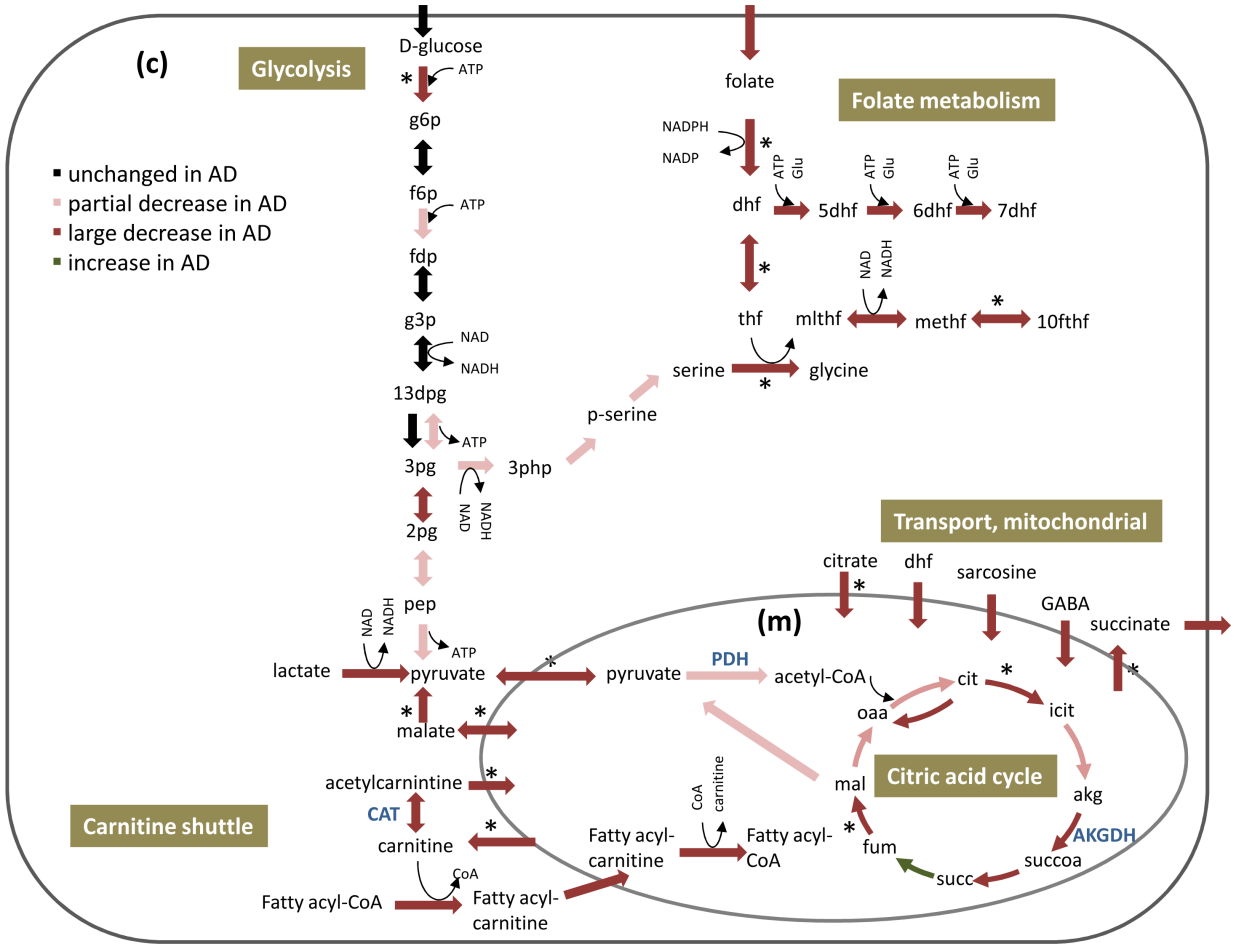
Key flux alterations in central metabolism predicted by iMAT for AD versus control states. The figure depicts the changes in energy metabolism in both cytosol (c) and mitochondrion (m). Several key enzymes whose activity was reported to decrease in AD patients are detailed in blue: pyruvate dehydrogenase (PDH), α-ketoglutarate dehydrogenase (AKGDH) and carnitine acetyltransferase (CAT). * Reactions whose activity changed significantly already at the transcription level.

Following the iMAT analysis, we examined which pathways had altered activity in AD versus the control (Table 1, Methods). This step was performed by employing flux variability analysis (FVA) [25] on the metabolic states inferred by iMAT for each of the AD vs. healthy states examined. The FVA analysis computes permissible flux intervals for each reaction, i.e., the minimal and maximal flux for each reaction that is yet consistent with the output of iMAT (Table S2). Then, by comparing the flux intervals of each reaction in its normal state and in AD, one can detect reactions whose activity is likely to be altered, and predict altered metabolic pathways. A number of pathways that were not manifested in a standard gene set enrichment analysis based on the gene expression alone were uncovered by our model-augmented analysis (Table 1, Figure 2). To bolster confidence in our results, we examined three sets of thresholds for determining when a given reaction is altered – that is, marking a ‘difference’ between its control and AD flux states. The pathways of carnitine shuttle, folate metabolism, and mitochondrial transport emerged robustly as the most over represented pathways with reduced flux activity in AD in all three cases (see Table S3). As expected, most of the fluxes across the network decrease in the disease, in accordance with the accepted notion of increased hypometabolism associated with AD.

**Table 1:** iMAT's predictions of metabolic pathways whose activity is significantly decreased in AD.

Pathway	p-value
Carnitine shuttle	3.53E^-18^
Folate metabolism*	3.78E^-13^
Transport, Mitochondrial*	4.77E^-11^
Fatty acid oxidation, peroxisome*	1.16E^-08^
Transport, Lysosomal	2.31E^-06^
Biotin metabolism	2.56E^-06^
N-glycan degradation	7.52E^-06^
IMP biosynthesis	2.21E^-05^
Valine, Leucine, and Isoleucine metabolism*	6.31E^-05^
Pyrimidine catabolism	1.13E^-03^
Arginine and Proline metabolism	1.17E^-03^
Phenylalanine metabolism	1.62E^-03^
Fatty acid metabolism*	4.76E^-03^

The table lists the pathways that are significantly decreased in AD according to iMAT predictions, as compared with the activity of control reactions. * Metabolic pathways that were significantly altered both in gene expression itself and in the model. All the results presented pass FDR of 0.05.

As mentioned earlier, differences between gene expression levels and enzyme flux activities as predicted by iMAT can indicate whether enzyme activity is post-transcriptionally increased or decreased compared to the original mRNA levels [18] (Figure 1). To test the metabolic descriptions we have obtained, we compared the predicted alterations in enzyme activities to the measured protein levels of these enzymes, according to proteomic data from temporal cortex of AD patients [28]. Reassuringly, we find significant overlap between predicted and experimentally determined differences in the levels of these proteins (hypergeometric p-value of 0.002). When focusing on reactions that are predicted by the AD model to be post-transcriptionally regulated, the calculated overlap p-value with the alterations reported in the proteomics data is 9.16e^−12^. Tryptophan metabolism was enriched among these reactions (p-value 2.5e^−4^, Table S4).

As a further testing of the metabolic descriptions obtained with the iMAT analysis, we identified the predicted alterations in metabolites exchange (secretion and uptake) between the cortex and biofluids in AD and normal patients, and compared our findings to experimentally determined metabolomic profiles in two patient sets in the CSF and the blood (Table S5). Our predicted alterations showed highly significant overlap with reported metabolomic alterations in both fluids (p-values: 8.4e^−26^ and 1.06e^−15^ in CSF and blood, respectively).

Finally, several key central metabolism enzymes whose flux has been predicted to decrease indeed have been reported to decrease their activity in AD [29]–[31]. These enzymes include PDH, AKGDH and cytochrome c oxidase (COX). All enzymes fluxes in this set are significantly decreased in the AD vs control predicted flux states, with p-values of 5e^−4^, 5e^−3^ and 2e^−7^, respectively.

The pathway predicted to decrease most significantly in AD is the carnitine shuttle, which, quite surprisingly, does not emerge in a standard gene expression enrichment test (Table 1). Carnitine shuttle is a carnitine dependent transport of fatty acids into the mitochondria for the production of energy via β-oxidation. Brain acyl-carnitines can function in synthesizing lipids, altering and stabilizing membrane composition, improving mitochondrial function, increasing antioxidant activity, and enhancing cholinergic neurotransmission [32]. A decreased activity of CAT has been measured in temporal cortex of AD patients [33] (and in our analysis as well - Figure 2), and it has been demonstrated that acetyl-carnitine administration can improve the cognitive performance in patients with mild AD [34].

Another pathway whose activity is predicted to decrease in AD is folate metabolism and the uptake of folate into the cell is also predicted to decrease (Figure 2 and Table S6). Experimental reports indicate a decrease of folate in the CSF of patients with AD [35]. Beyond the folate pathway itself, we find an overall dramatic decrease in the predicted activity of all reactions that have substrates of folate, dihydrofolate (DHF), or tetrahydrofolate (THF) (Figure S1).

Remarkably, the activity of reactions participating in metabolism of various neurotransmitters also decreased significantly in AD. This includes decreased uptake of acetylcholine and decreased activity of acetylcholinesterase, in accordance with reported decreases in levels and activity (respectively) in AD [36]; decreased secretion of norepinephrin, consistent with a previous metabolomic study showing its significant depletion in AD [37]; and decreased transport of 4-aminobutanoate (GABA) into the mitochondria.

### Prediction of metabolic biomarkers of AD

A major need in AD is the development of better biomarkers which can be read from accessible fluids, such as the blood [38]. As a first step in identifying potential biomarkers, we focus on predicting changes in extracellular transport reactions in the model (Methods, Table 2). A full list of metabolites with predicted secretion or uptake altered in disease is provided in Tables S6 and S7, respectively. As expected, most of the secretion and uptake fluxes of these biomarkers are predicted to decrease in AD.

**Table 2:** Metabolites whose secretion or uptake is markedly decreased in AD.

Metabolite	Decreased secretion/uptake
Succinate	secretion
Prostaglandin D2	secretion
D-Mannose	secretion
Sphingosylphosphorylcholine	uptake
Pentadecanoate	uptake
Heptadecanoate	uptake
D-Glucosamine	uptake

Among the biomarkers predicted here, succinate has been previously reported to significantly decrease in the CSF of AD patients [39]. Prostaglandin D2 (PGD2), whose secretion we predict to decrease as well (Table 2), is the most abundant prostaglandin in the brain and plays a role in regulation of sleep [40]. PGD2 mean level was found to slightly decrease in the CSF in AD patients; however, this change was not significant [41].

To predict plasma biomarkers in AD, we integrated recently reported gene expression data from blood leukocytes of AD and Mild Cognitive Impairment (MCI) patients [24] with the human metabolic model in a manner similar to that described previously with the cortical gene expression data (i.e. iMAT, see Methods), thus generating a metabolic description of these blood cells in AD. Next, we repeated the analyses detailed above and identified pathways that are enriched with altered reactions in blood leukocytes in AD and MCI (Figure S2). As evident, flux alterations in MCI and AD are quite similar. We found a significant overlap in metabolites we predicted to change in the blood (versus controls) with those reported in literature (P-value 1.15e^−18^, [42]–[44]). Table S8 lists our highest confidence blood biomarkers. Notably, we predict cholesterol to increase in the blood of AD patients as its secretion flux is predicted to increase. Altered cholesterol metabolism was suggested before in plasma of AD patients compared to MCI patients [45].

Intriguingly, several pathways whose activity is predicted to change in blood leukocytes of AD patients are also altered in the AD cortex. Among them, IMP biosynthesis was the only pathway that did not change in MCI blood leukocytes. Notably, the activities of IMP biosynthesis and fatty acid oxidation pathways increase in AD blood leukocytes but decrease in the cortex. Biomarkers predicted by both the cortical and the blood leukocytes analyses in AD are detailed in Table 3.

**Table 3:** Biomarkers predicted by both analyses of the cortex and the blood leukocytes in AD.

Metabolite name	Cortex	Blood
diacylglycerol**	secretion decrease	secretion increase
triacylglycerol**	uptake decrease	uptake increase
hyaluronan**	uptake decrease	secretion decrease*
prostaglandin D2**	secretion decrease*	secretion decrease
metanephrine	secretion decrease	secretion decrease

* Highly confident biomarkers (no overlap between flux intervals that is predicted for the control and AD).

** Biomarkers that are altered only in AD blood leukocytes and not in MCI.

### Prediction of drug targets by Metabolic Transformation Algorithm

**Figure 3:**
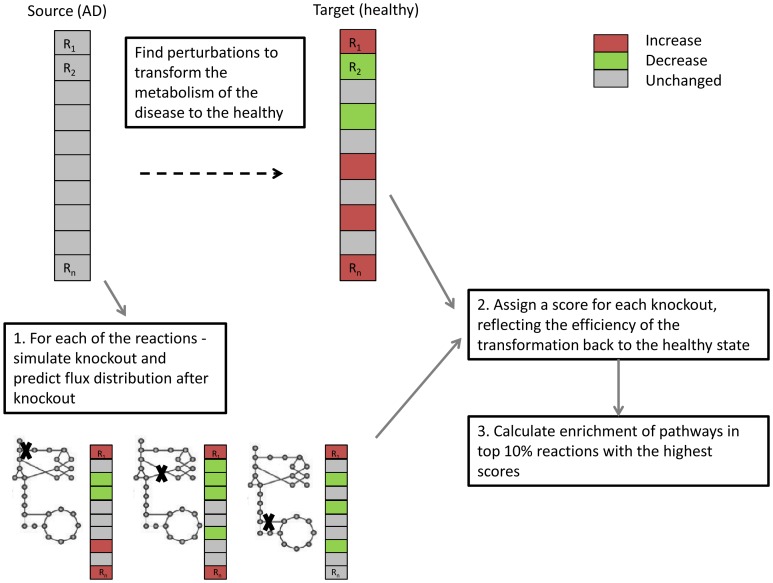
MTA workflow. MTA performs knockouts for each of the reactions in the metabolic model at the AD state (source), and assigns a score for each gene knockout reflecting the predicted extent by which this knockout may transform the metabolic state back to the healthy (target) state. Next, we aggregate the gene/reaction level predictions to identify the pathways whose knockout is predicted to be most successful in transforming the metabolic state as close as possible back to the healthy state.

Metabolic changes occur from the very earliest stages of AD. Although it is not known whether metabolism is the primary cause of the disease, these changes are extensive and may cause further feedback and exacerbation of neuronal death and disease progression [4]. Therefore, a drug that could reverse metabolic damage might have important therapeutic benefits. To predict candidate drug targets for AD we analyzed here the effects of metabolic gene knockouts using the human model. Our analysis is based on an algorithm termed Metabolic Transformation Algorithm (MTA) [26], which aims to identify gene perturbations that can transform metabolism from a given disease state back to a healthy one. This approach has already obtained promising results by identifying novel lifespan extending genes in yeast, which were then experimentally validated [26]. Here, we perform a systematic knockout of each gene in the human metabolic network (using the cortical gene expression data) and predict which knockouts will most likely transform the AD metabolic state back closer to the healthy one (Figure 3). The pathways enriched with reactions whose knockout is predicted by MTA to reverse AD's key metabolic alterations back closer to the healthy state are Vitamin D, nucleotides and Steroid metabolism (p-values 1.63e^−8^, 2.83e^−5^, 2.16e^−4^, respectively).

Vitamin D has been studied in recent years for its relation to cognitive performance and AD [9], [46], but its associations remain uncertain. Nevertheless, it has been increasingly recognized to play an active role in the nervous system [47], and a genome-wide association study of late-onset AD found evidence for involvement of the vitamin D receptor [47]. Steroid metabolism is another pathway we found enriched with predicted drug targets for AD. Intriguingly, the reaction that received the highest score within this pathway is 11-beta-hydroxysteroid dehydrogenase type 1 (11β-HSD1), an enzyme that catalyzes the intracellular regeneration of active glucocorticoids (i.e., cortisol and corticosterone). 11β-HSD1 knock-out mice have shown improved cognition, and 11β-HSD1 inhibitors improved memory in elderly men [48]. In general, steroids offer interesting therapeutic opportunities because of their varying roles in the nervous system: they regulate neurotransmitter systems, they promote the viability of neurons, and they influence cognitive processes [49].

Finally, a recent study by Searcy et al. showed that long-term Pioglitazone (PIO) treatment improved learning and decreased Aβ and tau deposits in a mouse model of AD [50]. Gene expression from the brains of these mice before and after the PIO treatment was also measured. For validation of the MTA predictions, we examined whether our set of top 10% knock-out predictions in humans is enriched with mouse orthologous genes whose expression was significantly decreased in the PIO treated mice with the improved phenotype. Encouragingly, we find such a significant overlap p-value of 0.025.

## Discussion

In the current study, we used genome scale metabolic modeling approaches to integrate gene expression measurements in the cortex of AD patients to address three key research questions: (1) what are the main metabolic alterations occurring in AD? (2) Which metabolites may serve as candidates for metabolic biomarkers of AD in the CSF and in the blood? And finally, (3) which metabolic genes may be silenced to most efficiently reverse the metabolic alterations observed in AD to a state of healthy aged matched controls?

We described the metabolic alterations in AD in both the cortex and blood leukocytes. The cortical analysis was based on a very large dataset of AD and control patients. However, for the analysis of the blood leukocyte we used a small dataset of gene expression that is publicly available (Methods) for comparison to the cortical predictions and between MCI and AD patients. A further analysis in the future utilizing richer gene expression datasets from blood cells of AD and MCI patients will aid to support this study's findings. Both analyses shared several pathways whose activity significantly increased in the blood and decreased in the brain, implying a possible compensation mechanism. Moreover, we predict biomarkers that are common to both analyses (i.e, cortex and blood), strengthening the potential of these metabolites as candidates for early diagnosis of AD. The MTA analysis yielded predictions of drug targets that may reverse the metabolic state of the disease back to the healthy one. Vitamin D and steroid metabolism appear in our analysis to be important in reversing the metabolic state in the disease. Furthermore, although it did not pass the FDR cutoff, our findings may hint to the importance of cholesterol in the pathogenesis of AD (P-value 0.015) and the potential value of keeping its levels in check [7]. The use of MTA for finding potential drug targets holds an advantage for finding drug candidates that act globally to reverse the entire metabolic network state to the healthy state, and thus may have lesser side effects.

Our analysis is in line with the common view that metabolism is overall decreased in AD. Several transport pathways appear throughout our analyses, further emphasizing the importance of metabolite transport in the disease. The predicted candidate biomarkers and drug targets that were discovered in this analysis may offer new metabolic leads for advancing the diagnosis of AD and its treatment. Hopefully, this work will motivate and guide future experimental studies geared at studying some of these leads.

## Supporting Information

Figure S1 **Maximal fluxes of reactions in which folate, DHF or THF act as substrates.**
(DOCX)

Figure S2 **Pathways enriched with reactions that are altered in AD and MCI blood leukocytes.**
(DOCX)

Table S1 **The list of genes that were defined as input for iMAT.**
(XLSX)

Table S2 **The list of reactions which their fluxes are predicted to alter in the disease.**
(XLSX)

Table S3 **Over represented pathways with altered reactions for different thresholds.**
(DOCX)

Table S4 **Tryptophan metabolism reactions which are PTR.**
(DOCX)

Table S5 **Metabolites level prediction in biofluids and experimental support.**
(DOCX)

Table S6 **Exchange reactions which their uptake fluxes alter in the cortex in AD.**
(DOCX)

Table S7 **Exchange reactions which their secretion fluxes alter in the cortex in AD.**
(DOCX)

Table S8 **Metabolites whose secretion or uptake are altered significantly in blood leukocytes in AD.**
(DOCX)
