# Supplementary material for: Integrating Transcriptomics with Metabolic Modeling Predicts Biomarkers and Drug Targets for Alzheimer's Disease
Source: PLoS One. 2014 Aug 15;9(8):e105383. doi: 10.1371/journal.pone.0105383 (PMC4134302; doi:10.1371/journal.pone.0105383)
Supplement: Figure S1 — Maximal fluxes of reactions in which folate, DHF or THF act as substrates. (DOCX) [file pone.0105383.s001.docx]

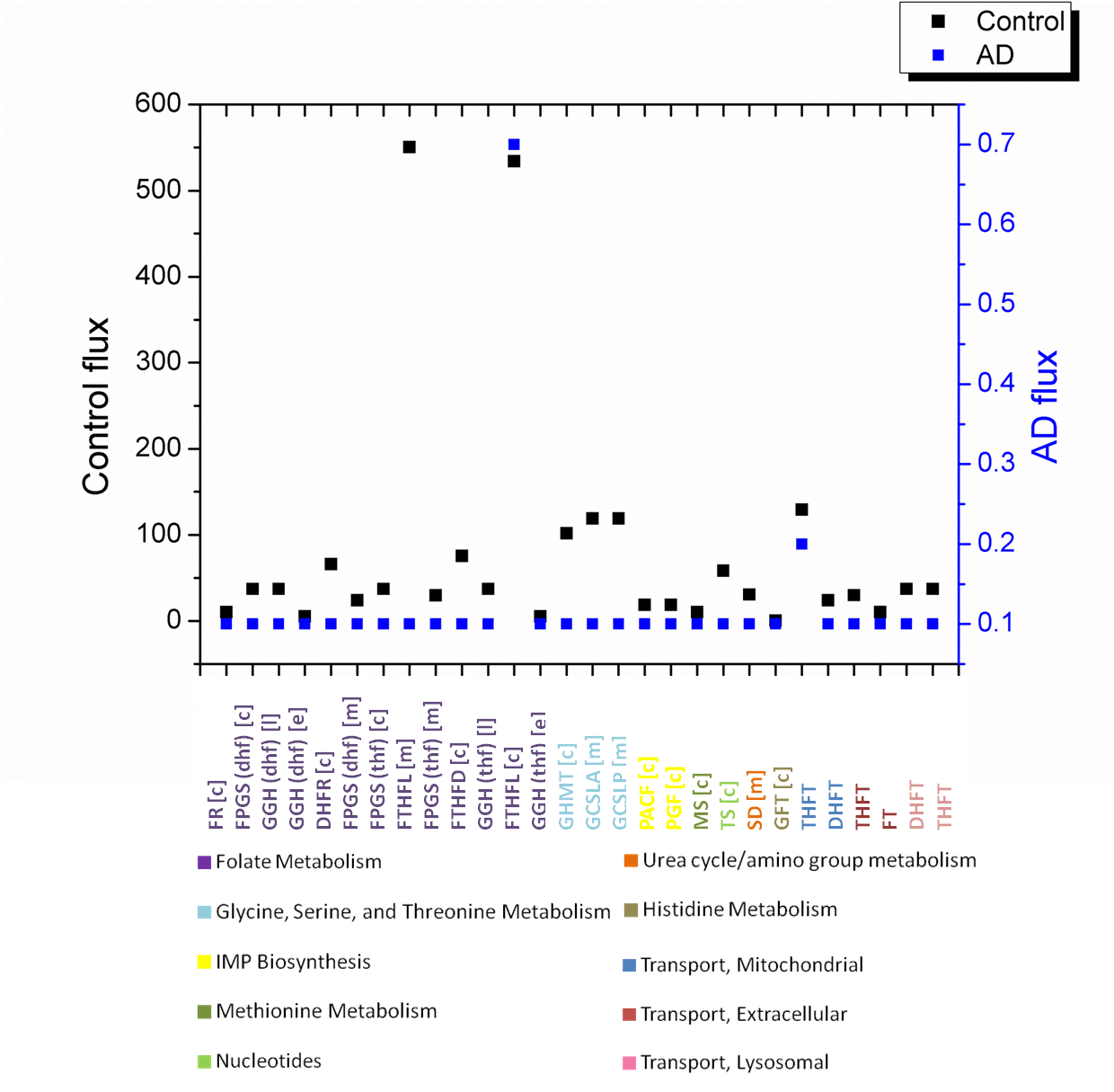


Figure S1: Maximal fluxes (absolute values) of reactions in which folate, DHF or THF act as substrates, as predicted in the control and AD models (black and blue squares, respectively). The enzymes catalyzing these reactions are detailed along the x axis together with their compartments and pertaining pathways. FR, folate reductase; FPGS, folylpolyglutamate synthetase; GGH, gamma-glutamyl hydrolase; DHFR, dihydrofolate reductase; FTHFL, formate-tetrahydrofolate ligase; FTHFD, formyltetrahydrofolate dehydrogenase; GHMT, glycine hydroxymethyltransferase; GCSLA, glycine-cleavage system (lipoamide); GCSLP, glycine-cleavage system (lipoylprotein); PACF, phosphoribosylaminoimidazole carboxamide formyltransferase; PGF, phosphoribosylglycinamide formyltransferase; MS, methionine synthase; TS, thymidylate synthase; SD, sarcosine dehydrogenase; GFT, glutamate formimidoyltransferase; THFT, 5,6,7,8-Tetrahydrofolate transport, DHFT, dihydrofolate transport; FT, folate transport. [c] cytosol, [m] mitochondrial [l] lysosomal, [e] extracellular.
