# Supplementary material for: Integrating Transcriptomics with Metabolic Modeling Predicts Biomarkers and Drug Targets for Alzheimer's Disease
Source: PLoS One. 2014 Aug 15;9(8):e105383. doi: 10.1371/journal.pone.0105383 (PMC4134302; doi:10.1371/journal.pone.0105383)
Supplement: Figure S2 — Pathways enriched with reactions that are altered in AD and MCI blood leukocytes. (DOCX) [file pone.0105383.s002.docx]

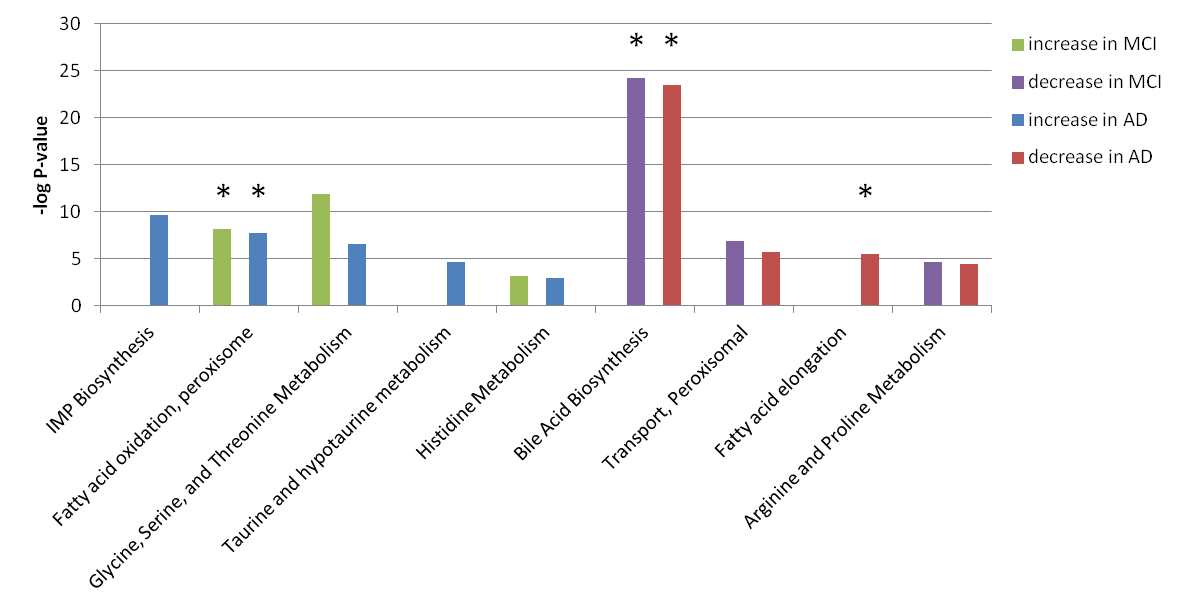


Figure S2: Pathways enriched with reactions that decrease or increase in AD and MCI blood leukocytes. * Metabolic pathways that were significantly altered both in standard gene set enrichment analysis and via a model-based analysis.
