# Supplementary material for: Integrating Transcriptomics with Metabolic Modeling Predicts Biomarkers and Drug Targets for Alzheimer's Disease
Source: PLoS One. 2014 Aug 15;9(8):e105383. doi: 10.1371/journal.pone.0105383 (PMC4134302; doi:10.1371/journal.pone.0105383)
Supplement: Table S3 — Over represented pathways with altered reactions for different thresholds. (DOCX) [file pone.0105383.s005.docx]

Table S3: Over represented pathways with altered reactions in AD, calculated for different thresholds

| threshold | 0.6 | 0.75 |
| --- | --- | --- |
| Enriched pathways | \| **'Carnitine shuttle'** \| \| --- \| \| **'Folate Metabolism'** \| \| 'Fatty acid oxidation' \| \| **'Transport, Mitochondrial'** \| \| 'Cholesterol Metabolism' \| \| 'Fatty acid oxidation, peroxisome' \| \| 'N-Glycan Biosynthesis' \| \| 'Biotin Metabolism' \| \| 'Valine, Leucine, and Isoleucine Metabolism' \| \| 'N-Glycan Degradation' \| \| 'Transport, Lysosomal' \| \| 'Pyrimidine Catabolism' \| \| 'IMP Biosynthesis' \| \| 'Glycerophospholipid Metabolism' \| \| 'R Group Synthesis' \| \| 'Aminosugar Metabolism ' \| \| 'Fructose and Mannose Metabolism' \| \| 'Triacylglycerol Synthesis' \| \| 'Glycolysis/Gluconeogenesis' \| \| 'Phenylalanine metabolism' \| \| 'Arginine and Proline Metabolism' \| \| 'Fatty Acid Metabolism' \| | \| **'Carnitine shuttle'** \| \| --- \| \| **'Transport, Mitochondrial'** \| \| **'Folate Metabolism'** \| \| 'Cholesterol Metabolism' \| \| 'Fatty acid oxidation, peroxisome' \| \| 'Valine, Leucine, and Isoleucine Metabolism' \| \| 'Transport, Lysosomal' \| \| 'Biotin Metabolism' \| \| 'N-Glycan Degradation' \| \| 'IMP Biosynthesis' \| \| 'Fatty acid oxidation' \| \| 'Pyrimidine Catabolism' \| \| 'Arginine and Proline Metabolism' \| \| 'Fatty Acid Metabolism' \| \| 'Phenylalanine metabolism' \| \| 'Glycerophospholipid Metabolism' \| |
