# Supplementary material for: Integrating Transcriptomics with Metabolic Modeling Predicts Biomarkers and Drug Targets for Alzheimer's Disease
Source: PLoS One. 2014 Aug 15;9(8):e105383. doi: 10.1371/journal.pone.0105383 (PMC4134302; doi:10.1371/journal.pone.0105383)
Supplement: Table S4 — Tryptophan metabolism reactions which are PTR. (DOCX) [file pone.0105383.s006.docx]

Table S4: Tryptophan metabolism reactions which are PTR

| 5-Hydroxytryptamine:oxygen oxidoreductase(deaminating)  (flavin-containing) | H2O + O2 + Serotonin => 5-Hydroxyindoleacetaldehyde + Hydrogen peroxide + Ammonium |
| --- | --- |
| Tryptamine:oxygen oxidoreductase(deaminating)  (flavin-containing) | H2O + O2 + Tryptamine => Hydrogen peroxide + Indole-3-acetaldehyde + Ammonium |
| 5-Hydroxykynurenamine:oxygen oxidoreductase(deaminating)  (flavin-containing) | 5-Hydroxykynurenamine + O2 => 4,6-Dihydroxyquinoline + Hydrogen peroxide + Ammonium |
| aldehyde dehydrogenase (indole-3-acetaldehyde, NAD) | H2O + Indole-3-acetaldehyde + Nicotinamide adenine dinucleotide => 2H+ + Indole-3-acetate + Nicotinamide adenine dinucleotide - reduced |
| 3-Hydroxykynurenamine:oxygen oxidoreductase(deaminating)  (flavin-containing) | 3-Hydroxykynurenamine + O2 => 4,8-Dihydroxyquinoline + Hydrogen peroxide + Ammonium |
| 5-Hydroxyindoleacetaldehyde:NAD+ oxidoreductase | 5-Hydroxyindoleacetaldehyde + H2O + Nicotinamide adenine dinucleotide => 5-Hydroxyindoleacetate + 2H+ + Nicotinamide adenine dinucleotide - reduced |
