# Supplementary material for: Integrating Transcriptomics with Metabolic Modeling Predicts Biomarkers and Drug Targets for Alzheimer's Disease
Source: PLoS One. 2014 Aug 15;9(8):e105383. doi: 10.1371/journal.pone.0105383 (PMC4134302; doi:10.1371/journal.pone.0105383)
Supplement: Table S5 — Metabolites level prediction in biofluids and experimental support. (DOCX) [file pone.0105383.s007.docx]

Table S5: Metabolites level prediction in biofluids and experimental support in the CSF and the blood

| **Metabolite name** | Predicted to change in biofluids in AD model | Experimental support in the CSF | Experimental support in the blood |
| --- | --- | --- | --- |
| myo-Inositol | - | [^1^](#_ENREF_1) | [^2^](#_ENREF_2) |
| 3,4-Dihydroxy-L-phenylalanine | - |  |  |
| L-Arginine | + |  | [^3^](#_ENREF_3) |
| D-Mannose | + |  |  |
| D-Fructose | - | [^1^](#_ENREF_1) |  |
| Choline | - | [^1^](#_ENREF_1) |  |
| D-Lactate | - | [^1^](#_ENREF_1) |  |
| Uridine | - |  |  |
| L-Leucine | + | [^3^](#_ENREF_3)^,^ [^4^](#_ENREF_4) |  |
| D-Glucose | - | [^1^](#_ENREF_1) |  |
| L-Phenylalanine | + | [^1^](#_ENREF_1) |  |
| Urea | - | [^1^](#_ENREF_1) |  |
| L-Cysteine | - |  | [^5^](#_ENREF_5) |
| L-Glutamine | - | [^1^](#_ENREF_1)^,^ [^4^](#_ENREF_4) | [^5^](#_ENREF_5) |
| L-Serine | - | [^4^](#_ENREF_4) | [^5^](#_ENREF_5) |
| Citrate | - | [^1^](#_ENREF_1) |  |
| (S)-Glycerate | - | [^1^](#_ENREF_1) |  |
| Creatine | + | [^3^](#_ENREF_3) | [^3^](#_ENREF_3) |
| Phosphate | - | [^1^](#_ENREF_1) |  |
| L-Tryptophan | - | [^1^](#_ENREF_1)^,^ [^6^](#_ENREF_6) | [^5^](#_ENREF_5) |
| Urate | - | [^1^](#_ENREF_1)^,^ [^6^](#_ENREF_6) |  |
| Cholesterol | - | [^1^](#_ENREF_1) |  |
| Dopamine | - |  | [^5^](#_ENREF_5) |
| L-Threonine | + |  |  |
| L-Carnitine | - | [^1^](#_ENREF_1) |  |
| L-Isoleucine | + | [^7^](#_ENREF_7) |  |
| L-Lysine | + | [^7^](#_ENREF_7) |  |
| L-Valine | + | [^4^](#_ENREF_4) |  |
| L-Methionine | - | [^4^](#_ENREF_4)^,^ [^6^](#_ENREF_6) | [^4^](#_ENREF_4) |
| L-Ascorbate | + |  | [^8^](#_ENREF_8) |
| Pyruvate | - | [^9^](#_ENREF_9) |  |
| Ornithine | + | [^1^](#_ENREF_1) |  |
| Taurine | - | [^1^](#_ENREF_1)^,^ [^4^](#_ENREF_4) |  |
| L-Tyrosine | - | [^4^](#_ENREF_4)^,^ [^6^](#_ENREF_6) | [^5^](#_ENREF_5) |
| L-Alanine | - | [^1^](#_ENREF_1)^,^ [^4^](#_ENREF_4) | [^4^](#_ENREF_4) |
| (R)-Pantothenate | - | [^1^](#_ENREF_1) |  |
| Histamine | + | [^1^](#_ENREF_1) |  |
| Folic acid | + | [^7^](#_ENREF_7) | [^10^](#_ENREF_10) |
| Glycine | - | [^11^](#_ENREF_11) | [^5^](#_ENREF_5) |
| L-Proline | + | [^7^](#_ENREF_7) |  |
| L-Cystine | - | [^6^](#_ENREF_6) | [^5^](#_ENREF_5) |
| Norepinephrine | + | [^6^](#_ENREF_6) |  |
| 5-Hydroxytryptophan | - | [^6^](#_ENREF_6) |  |
| Alpha tocopherol | - | [^6^](#_ENREF_6) |  |
| Guanosine | - | [^6^](#_ENREF_6) |  |
| Serotonin | - | [^6^](#_ENREF_6) |  |
| Glutathione(reduced) | - | [^6^](#_ENREF_6) |  |
| Hypoxanthine | - | [^6^](#_ENREF_6) |  |
| 4-Hydroxyphenyllactic acid | - | [^6^](#_ENREF_6) |  |
| Glutathione (oxidized) | - | [^6^](#_ENREF_6) |  |
| succinate | + | [^9^](#_ENREF_9) |  |
| acetoacetate | - | [^9^](#_ENREF_9) |  |
| Histidine | - | [^4^](#_ENREF_4) | [^5^](#_ENREF_5) |
| Ribitol | - | [^2^](#_ENREF_2) | [^2^](#_ENREF_2) |
| thiamine | - | [^12^](#_ENREF_12) |  |
| thiamine monophosphate | - | [^12^](#_ENREF_12) | [^12^](#_ENREF_12) |
| acetylcholine | + | [^13^](#_ENREF_13) |  |
| prostaglandin E2 | - |  |  |
| prostaglandin D2 | + |  |  |
| prostaglandin F2alpha | - | [^14^](#_ENREF_14) |  |
| Aspartic acid | - |  | [^5^](#_ENREF_5) |
| L-thyroxin | - |  | [^15^](#_ENREF_15) |
| Triiodothyronine | - | [^15^](#_ENREF_15) | [^15^](#_ENREF_15) |
| phosphatidylcholine | + |  |  |
| Glutamic acid | - |  | [^5^](#_ENREF_5) |
| GABA | - |  | [^5^](#_ENREF_5) |

**References**

1. Czech, C. et al. Metabolite profiling of Alzheimer's disease cerebrospinal fluid. *PloS one* **7**, e31501 (2012).

2. Shetty, H.U., Holloway, H.W. & Schapiro, M.B. Cerebrospinal fluid and plasma distribution of myo-inositol and other polyols in Alzheimer disease. *Clinical chemistry* **42**, 298-302 (1996).

3. Trushina, E., Dutta, T., Persson, X.M., Mielke, M.M. & Petersen, R.C. Identification of altered metabolic pathways in plasma and CSF in mild cognitive impairment and Alzheimer's disease using metabolomics. *PloS one* **8**, e63644 (2013).

4. Basun, H. et al. Amino acid concentrations in cerebrospinal fluid and plasma in Alzheimer's disease and healthy control subjects. *Journal of neural transmission. Parkinson's disease and dementia section* **2**, 295-304 (1990).

5. Fonteh, A.N., Harrington, R.J., Tsai, A., Liao, P. & Harrington, M.G. Free amino acid and dipeptide changes in the body fluids from Alzheimer's disease subjects. *Amino acids* **32**, 213-224 (2007).

6. Kaddurah-Daouk, R. et al. Metabolomic changes in autopsy-confirmed Alzheimer's disease. *Alzheimer's & dementia : the journal of the Alzheimer's Association* **7**, 309-317 (2011).

7. Wishart, D.S. et al. HMDB: a knowledgebase for the human metabolome. *Nucleic acids research* **37**, D603-610 (2009).

8. McGrath, L.T. et al. Increased oxidative stress in Alzheimer's disease as assessed with 4-hydroxynonenal but not malondialdehyde. *QJM : monthly journal of the Association of Physicians* **94**, 485-490 (2001).

9. Redjems-Bennani, N. et al. Abnormal substrate levels that depend upon mitochondrial function in cerebrospinal fluid from Alzheimer patients. *Gerontology* **44**, 300-304 (1998).

10. McCaddon, A. et al. Alzheimer's disease and total plasma aminothiols. *Biological psychiatry* **53**, 254-260 (2003).

11. D'Aniello, A. et al. Amino acids and transaminases activity in ventricular CSF and in brain of normal and Alzheimer patients. *Neuroscience letters* **388**, 49-53 (2005).

12. Molina, J.A. et al. Cerebrospinal fluid levels of thiamine in patients with Alzheimer's disease. *J Neural Transm* **109**, 1035-1044 (2002).

13. Tohgi, H., Abe, T., Hashiguchi, K., Saheki, M. & Takahashi, S. Remarkable reduction in acetylcholine concentration in the cerebrospinal fluid from patients with Alzheimer type dementia. *Neuroscience letters* **177**, 139-142 (1994).

14. Montine, T.J. et al. Elevated CSF prostaglandin E2 levels in patients with probable AD. *Neurology* **53**, 1495-1498 (1999).

15. Johansson, P. et al. Reduced cerebrospinal fluid level of thyroxine in patients with Alzheimer's disease. *Psychoneuroendocrinology* **38**, 1058-1066 (2013).
