# Supplementary material for: Integrating Transcriptomics with Metabolic Modeling Predicts Biomarkers and Drug Targets for Alzheimer's Disease
Source: PLoS One. 2014 Aug 15;9(8):e105383. doi: 10.1371/journal.pone.0105383 (PMC4134302; doi:10.1371/journal.pone.0105383)
Supplement: Table S6 — Exchange reactions which their uptake fluxes alter in the cortex in AD. (DOCX) [file pone.0105383.s008.docx]

Table S6: Exchange reactions which their uptake fluxes alter significantly in the cortex in AD (overlap <=0.1)

| Exchange reaction | Decreased/increased | pathways involved |
| --- | --- | --- |
| keratan sulfate I | decreased | Keratan sulfate biosynthesis & degradation |
| paclitaxel * | decreased | CYP Metabolism |
| UTP | decreased | Nucleotides;Transport, Mitochondrial |
| heptadecanoate * | decreased | Fatty acid activation; Carnitine shuttle |
| 1-alkyl 2-acteylglycerol 3-phosphocholine | decreased | Glycerophospholipid Metabolism |
| L-Leucine | decreased | Valine, Leucine, and Isoleucine Metabolism;Transport, Mitochondrial |
| Nicotinamide | decreased | NAD Metabolism |
| D-Glucosamine* | decreased | Aminosugar Metabolism |
| Nicotinate | decreased | NAD Metabolism |
| AMP | decreased | Nucleotides and many others |
| heptaglutamyl folate | decreased | Folate Metabolism; Transport, Mitochondrial |
| Sarcosine | decreased | Glycine, Serine, and Threonine Metabolism; Urea cycle/amino group metabolism; Transport, Peroxisomal, Mitochondrial |
| GTP | decreased | Nucleotides and many others |
| Acetylcholine | decreased | Glycerophospholipid Metabolism |
| Biocytin | decreased | Biotin Metabolism; Transport, Nuclear |
| Folate | decreased | Folate metabolism |
| L-Threonine | decreased | Glycine, Serine, and Threonine Metabolism |
| Pentadecanoate* | decreased | Fatty acid activation; Carnitine shuttle |
| IDP | decreased | Nucleotides; Transport, Nuclear |
| 10-formyltetrahydrofolate-[Glu](7) | decreased | Folate Metabolism; Transport, Mitochondrial |
| D-Ribose | decreased | Pentose Phosphate Pathway |
| CMP | decreased | Nucleotides and many others |
| Ornithine | decreased | Urea cycle/amino group metabolism |
| 5-Methyltetrahydrofolate | decreased | Folate Metabolism; Methionine Metabolism |
| Histamine | decreased | Histidine Metabolism |
| 5-Formyltetrahydrofolate | decreased | Folate Metabolism |
| ebastine | decreased | CYP Metabolism |
| Sphingosylphosphorylcholine* | decreased | Sphingolipid Metabolism |

*no overlap between control flux interval and AD flux interval
