# Supplementary material for: Integrating Transcriptomics with Metabolic Modeling Predicts Biomarkers and Drug Targets for Alzheimer's Disease
Source: PLoS One. 2014 Aug 15;9(8):e105383. doi: 10.1371/journal.pone.0105383 (PMC4134302; doi:10.1371/journal.pone.0105383)
Supplement: Table S7 — Exchange reactions which their secretion fluxes alter in the cortex in AD. (DOCX) [file pone.0105383.s009.docx]

Table S7: Exchange reactions which their secretion fluxes alter significantly in the cortex in AD (overlap <=0.1)

| Exchange reaction | Decreased/increased | pathways involved |
| --- | --- | --- |
| hydroxylated ebastine | decreased | CYP Metabolism |
| Metanephrine | decreased | Tyrosine metabolism |
| D-Mannose * | decreased | Fructose and Mannose Metabolism N-Glycan Degradation and Biosynthesis, Transport  (Lysosomal, Endoplasmic Reticular, Extracellular, Golgi Apparatus) |
| alpha-N-Phenylacetyl-L-glutamine | decreased | Phenylalanine metabolism |
| 3-Methylimidazoleacetic acid | decreased | Histidine Metabolism |
| Nitric oxide | decreased | Arginine and Proline Metabolism |
| D-3-Amino-isobutanoate | decreased | Pyrimidine Catabolism |
| 2,4 dihydroxy nitrophenol | decreased | CYP Metabolism |
| Biotin | decreased | Biotin Metabolism, Transport, Mitochondrial, Nuclear |
| Thromboxane A2 | decreased | Eicosanoid Metabolism |
| hydroxylated taxol | decreased | CYP Metabolism |
| 10-Formyltetrahydrofolate | decreased | Folate Metabolism, IMP Biosynthesis, Transport (Mitochondrial, Lysosomal) |
| cholesterol ester | decreased | Fatty Acid Metabolism |
| Succinate* | decreased | Citric Acid Cycle, Valine, Leucine, and Isoleucine Metabolism, Lysine Metabolism, Glutamate metabolism,  Fatty acid oxidation, peroxisome,  Arginine and Proline Metabolism,  Transport(Mitochondrial, Peroxisomal) |
| 1-alkyl 2-lysoglycerol 3-phosphocholine | decreased | Glycerophospholipid Metabolism |
| 1-Methylnicotinamide | decreased | NAD Metabolism |
| 7,8-Dihydrofolate | decreased | Folate Metabolism; Transport (Mitochondrial, Lysosomal),  Nucleotides |
| IMP | decreased | Nucleotides, Salvage Pathway, Purine Catabolism, IMP Biosynthesis |
| Prostaglandin D2* | decreased | Eicosanoid Metabolism |
| Thiamin triphosphate | decreased | Thiamine Metabolism |
| Sphingosylphosphorylcholine* | increased | Sphingolipid Metabolism |

*no overlap between control flux interval and AD flux interval
