# Supplementary material for: Integrating Transcriptomics with Metabolic Modeling Predicts Biomarkers and Drug Targets for Alzheimer's Disease
Source: PLoS One. 2014 Aug 15;9(8):e105383. doi: 10.1371/journal.pone.0105383 (PMC4134302; doi:10.1371/journal.pone.0105383)
Supplement: Table S8 — Metabolites whose secretion or uptake are altered significantly in blood leukocytes in AD. (DOCX) [file pone.0105383.s010.docx]

Table S8: Metabolites whose secretion or uptake are altered significantly in blood leukocytes in AD

| Metabolite | Secretion/  Uptake | decrease/  increase | MCI/ AD |
| --- | --- | --- | --- |
| diacylglycerol | Secretion | increase | AD |
| Sulfate | Secretion | increase | MCI ,AD |
| nC22:6 | Secretion | increase | MCI ,AD |
| docosa-4,7,10,13,16-pentaenoic acid (n-6) | Secretion | increase | MCI ,AD |
| Timnodonic acid | Secretion | increase | MCI ,AD |
| Formate | Secretion | increase | MCI ,AD |
| L-Serine | Secretion | increase | MCI ,AD |
| Deoxyribose | Secretion | increase | AD |
| Cholesterol | Secretion | increase | AD |
| Glycine | Secretion | increase | MCI ,AD |
| Sphinganine 1-phosphate | Secretion | increase | MCI ,AD |
| Metanephrine | Secretion | decrease | MCI ,AD |
| Adrenaline | Secretion | decrease | MCI ,AD |
| Chenodeoxyglycocholate* | Secretion | decrease | MCI ,AD |
| Tyramine O-sulfate | Secretion | decrease | AD |
| Hyaluronan* | Secretion | decrease | AD |
| 4,17 dihydroxy estradiol* | Secretion | decrease | AD |
| N-Acetyl-D-glucosamine | Secretion | decrease | AD |
| 25-Hydroxyvitamin D3* | Secretion | decrease | AD |
| Taurine | Uptake | decrease | MCI ,AD |
| Taurocholic acid | Secretion | decrease | MCI ,AD |
| L-Tyrosine | Uptake | decrease | AD |
| chitin | Uptake | decrease | AD |

* no overlap between AD and control flux intervals.
